# Manufacturing of dental pulp cell-based products from human third molars: current strategies and future investigations

**DOI:** 10.3389/fphys.2015.00213

**Published:** 2015-08-06

**Authors:** Maxime Ducret, Hugo Fabre, Olivier Degoul, Gianluigi Atzeni, Colin McGuckin, Nico Forraz, Brigitte Alliot-Licht, Frédéric Mallein-Gerin, Emeline Perrier-Groult, Jean-Christophe Farges

**Affiliations:** ^1^Laboratoire de Biologie Tissulaire et Ingénierie thérapeutique, UMR5305 Centre National de la Recherche Scientifique/Université Claude Bernard Lyon 1Lyon, France; ^2^Faculté d'Odontologie, Université de Lyon, Université Claude Bernard Lyon 1Lyon, France; ^3^Hospices Civils de Lyon, Service de Consultations et Traitements DentairesLyon, France; ^4^CTI-BIOTECH, Cell Therapy Research InstituteMeyzieu, France; ^5^Faculté d'Odontologie, Institut National de la Santé et de la Recherche Médicale UMR1064, Centre de Recherche en Transplantation et Immunologie, Université de NantesNantes, France

**Keywords:** human dental pulp, stem cells, tissue engineering, immunophenotyping, expansion, cryopreservation, good manufacturing practices, cell-based medicinal products

## Abstract

In recent years, mesenchymal cell-based products have been developed to improve surgical therapies aimed at repairing human tissues. In this context, the tooth has recently emerged as a valuable source of stem/progenitor cells for regenerating orofacial tissues, with easy access to pulp tissue and high differentiation potential of dental pulp mesenchymal cells. International guidelines now recommend the use of standardized procedures for cell isolation, storage and expansion in culture to ensure optimal reproducibility, efficacy and safety when cells are used for clinical application. However, most dental pulp cell-based medicinal products manufacturing procedures may not be fully satisfactory since they could alter the cells biological properties and the quality of derived products. Cell isolation, enrichment and cryopreservation procedures combined to long-term expansion in culture media containing xeno- and allogeneic components are known to affect cell phenotype, viability, proliferation and differentiation capacities. This article focuses on current manufacturing strategies of dental pulp cell-based medicinal products and proposes a new protocol to improve efficiency, reproducibility and safety of these strategies.

## Introduction

Over the two last decades, mesenchymal stromal cells (MSC) have been intensely studied due to their potential clinical applicability to treat tissue and organ defects resulting from diseases, trauma or aging (Caplan, 1991). Their use has been proposed to repair and regenerate human mesenchymal tissues, alone or combined to scaffolds and/or morphogenic molecules (Langer and Vacanti, 1993). Bone marrow and adipose tissue are conventional sources of MSC, but invasive cell collection protocols, frequent use of general anesthesia and risk of morbidity at the collection site have stimulated the search for alternative tissues (Huang et al., 2009; Zuk, 2010; Davies et al., 2015). Third molars are frequently removed for therapeutic reasons and the connective tissue it contains, the dental pulp, can be easily recovered. They are now considered a valuable source of MSC for tissue repair and regeneration (Mayo et al., 2014). In this context, numerous investigators have attempted to obtain clinical-grade dental pulp stem/progenitor cells (DPSC) from these teeth. However, most manufacturing procedures reported so far may not be totally satisfactory, since they may alter the biological properties of the cells and the quality of the derived cell-based products (Ménard and Tarte, 2013). If such procedures are currently permitted by European and American regulation authorities, further studies are necessary to develop more efficient, reproducible, safe and standardized manufacturing processes of dental pulp cell-based medicinal products (Tirino and Papaccio, 2012; Albuquerque et al., 2014; Eubanks et al., 2014; Huang and Garcia-Godoy, 2014; La Noce et al., 2014; Nakashima and Iohara, 2014).

Dental pulp mesenchymal cells have been successfully used to regenerate human craniofacial bone (d'Aquino et al., 2009; Giuliani et al., 2013). However, these studies were performed in the absence of defined, universally accepted protocols for large-scale, clinical-grade production of DPSC (Fekete et al., 2012). This point is important in the light of recent reports indicating moderate, irreproducible and non-suitable benefits of therapies performed with various sources of MSC (Allison, 2009; Tyndall, 2011; Daley, 2012). These results were explained in part by the fact that cell performances are affected by cell isolation and expansion conditions and indicate the need for optimized and standardized procedures for MSC-based products' manufacturing (Allison, 2009; Pacini, 2014). The European Union (EU) and United States (US) have established classifications and recommended guidelines for manufacturing MSC-based products. In Europe, MSC are defined as “cell therapy products” and referred to as Advanced Therapy Medicinal Products (ATMP) (European Regulation 1394/2007). ATMP are considered Cell-Based Medicinal Products (CBMP) when containing living cells or tissues. CBMP are “medicinal products presented as having properties for, or used in or administered to, human beings with a view to treating, preventing or diagnosing a disease in which the pharmacological, immunological or metabolic actions are carried out by cells or tissues” (Schneider et al., 2010; Pacini, 2014). DPSC belong to this category and can be referred to as Dental Pulp (DP)-CBMP. In the US, DPSC are considered as Human Cells, Tissues or cellular and tissue-based Products (HCT/Ps) (Code of Federal Regulation (CFR) Title 21 CFR 1271). They are classified in two categories: (1) products that are “minimally manipulated” and used clinically in a homologous manner, and (2) products that are either “more than minimally manipulated” or used in a non-homologous manner. A cell-based product is considered as being “more than minimally manipulated” when the inherent biological characteristics of the cells have been significantly altered (Pacini, 2014).

**Figure 1:**
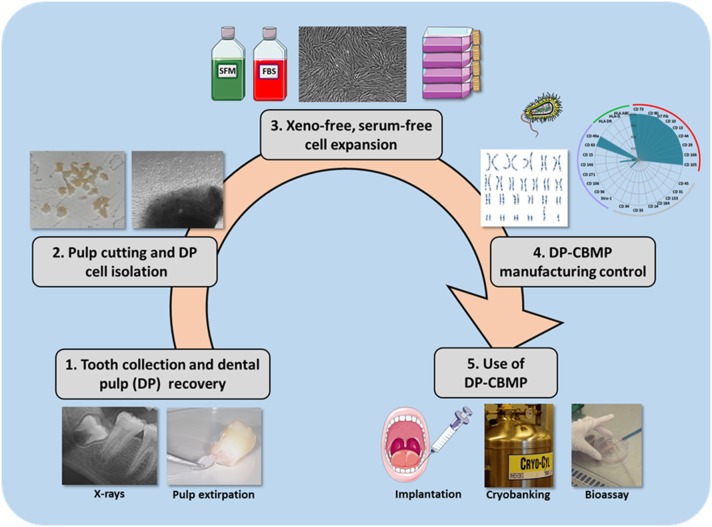
**Standardization of the DP-CBMP manufacturing process**. It requires five major steps: tooth selection and use of the easiest technique for pulp recovery (Step 1), HDPC isolation with fast, safe and less expensive procedures (Step 2), cell expansion in defined, serum-free culture conditions with xeno-free reagents (Step 3), advanced monitoring and control of DP-CBMP manufacturing (Step 4), use of clinical-grade DP-CMBP, for immediate implantation, cryobanking or development of a bioassay (Step 5).

Production and delivery of MSCs should be made in accordance with European Good Manufacturing Practices (GMP), whereas, in the US, it must comply with Current Good Tissue Practice requirements (GTP) (Fekete et al., 2012; Kellathur and Lou, 2012; Sensebé et al., 2013). GMP/GTP require many quality controls regarding donor eligibility, sample recovery, label, transport and receipt, process and storage, laboratory equipment, supplies and reagents, cell-based product distribution to recipient patients and documentation that must be maintained by the handler (Alici and Blomberg, 2010; Abou-El-Enein et al., 2013; Sensebé et al., 2013; Wuchter et al., 2015). These controls make GMP/GTP procedures long and costly, and further studies are encouraged to develop shorter, less expensive and more standardized procedures for DP-CBMP manufacturing (Albuquerque et al., 2014; Eubanks et al., 2014; Huang and Garcia-Godoy, 2014; La Noce et al., 2014; Nakashima and Iohara, 2014; Hilkens et al., 2015). In the present paper, we will firstly review current international guidelines regarding the five manufacturing steps of DP-CBMP (Figure 1), and then we will highlight the drawbacks and potential risks of actual strategies. Finally we will propose modifications of the protocols intended to increase the efficiency, reproducibility and safety of these strategies, from tooth extraction to the harvest of clinical-grade DP-CMBP.

## Teeth collection and pulp tissue recovery

Since the discovery of DPSC by Gronthos et al. (2000), numerous papers have reported the isolation of stem/progenitor cells from the dental pulp of human third molars. However, since there are no rules specifying the best tooth development stages for pulp cell collection, teeth were collected in patients of various ages and therefore at various developmental stages. It also greatly impairs the interpretation and comparison of the experimental results.

Transport from the operating block to the laboratory notably requires a medium that does not affect cell viability. It was previously shown that DPSC remain viable up for 5 days when extracted teeth are maintained in phosphate-buffered saline (PBS) (Perry et al., 2008; Woods et al., 2009) and this time is more than enough for the transport of samples to the laboratory and pulp recovery.

## Dental pulp cell isolation and enrichment

After dental pulp recovery, two options are possible for isolating dental pulp cells: enzymatic dissociation and explant culture. Enzymatic dissociation consists of digesting the pulp tissue with collagenase and dispase enzymes to liberate the cells that are then plated on culture dishes. However, a growing number of authors consider that enzymatic dissociation is not adapted to medicinal manufacturing, owing to its putative consequences on cell phenotype and properties (Shah et al., 2013; Busser et al., 2014; Ohnuma et al., 2014). In addition, tissues and cells exposed to collagenase are considered “more than minimally manipulated” by FDA [Code of Federal Regulation (CFR) Title 21 CFR 1271] and potentially require the use of pharmaceutical grade manufactured enzymes, which significantly increases the scale-up costs. By contrast, cell isolation by explant culture increasingly appears easier, faster, safer, less expensive and more in line with GMP guidelines to obtain clinical-grade amounts of MSC (Hilkens et al., 2013). It is based on the growth of cells out of tissue fragments (explants) that are plated on culture dishes. It recently allowed for efficient recovery of human adipose or Wharton jelly stem/progenitor cells in serum-free, xeno-free medium conditions (Busser et al., 2014; Swamynathan et al., 2014). Additionally, explant-derived DPSC display similar or enhanced differentiation abilities compared with cells from dissociated tissue (Spath et al., 2010; Hilkens et al., 2013).

Cell selection by sorting methods has been proposed to enrich the cultured cell population in stem/progenitor cells based on their expression of specific surface markers such as CD34, CD184, Stro-1, CD146, CD271, and MSCA-1 (Shi and Gronthos, 2003; d'Aquino et al., 2009; Waddington et al., 2009; Yu et al., 2010; Jiang et al., 2012; Tomlinson et al., 2015). However, the use of such a procedure is today limited by the complexity of the technique and the prohibitive cost (Kawashima, 2012; Nakashima and Iohara, 2014). Besides, multiplying steps and using additional reagents increase the risk of microbial contamination and the difficulty to obtain CBMP in GMP conditions. The same reservation can be made regarding the use of biophysical markers that have been found relevant to isolate MSC in an easier and more predictable way than biochemical markers (Lee et al., 2014).

## Dental pulp cell culture and expansion

Among other factors, the composition of the culture medium and the presence of a coating material on the culture dish may influence the nature and the quality of the final CBMP and therefore the clinical results (Lopez-Cazaux et al., 2006; Majd et al., 2009; Jung et al., 2012; Pisciotta et al., 2012; Pacini, 2014). Currently, CBMP manufacturing under GMP procedures recommends the use of xeno-free materials and reagents to prevent the risk of viral, bacterial, fungal and prion contamination, and the possible induction of immunizing effects in the final recipient. Additionally, industrial production is responsible for frequent batch-to-batch serum variability and the serum itself can promote early cell differentiation (Mannello and Tonti, 2007; Jung et al., 2012). For these reasons, the supplementation of the cell culture medium with xeno- or allogeneic products should be limited to “cases for which a valid alternative cannot be found” (European Regulation 1394/2007). Today, the development of xeno-free, serum-free, defined media, able to rapidly expand stem/progenitor cells without impairing their differentiation capabilities, represents a major objective for the standardization of DP-CBMP production (Tekkatte et al., 2011; Jung et al., 2012; Bonnamain et al., 2013; Carvalho et al., 2013). Multiple passages are often necessary to obtain a clinical-scale amount of cells, but they may lead to a slow-down of the proliferation rate, progressive cell senescence and loss of multipotentiality that prevent future cell differentiation (Baxter et al., 2004; Bork et al., 2010; Yu et al., 2010; Sensebé et al., 2013). In our culture conditions, cell doubling times remained constant from P1 to P4 (≈40 h) and we calculated that more than 25.10^7^ cells could be theoretically obtained after four passages with one dental pulp, which is likely to be a sufficient cell number for one pulp regeneration, bone socket filling, or for localized periodontal treatment (Kaigler et al., 2013; Albuquerque et al., 2014).

## DP-CBMP manufacturing control

DP-CBMP manufacturing requires advanced quality controls of the safety, identity and efficacy of the final product (Wang et al., 2005; Sensebé et al., 2013). Since CBMP cannot undergo sterilization before implantation, the absence of bacteria, virus, fungi and prion contamination has to be checked. The presence of endotoxin must also be tested to prevent immune reactions in the recipient patient. Long-term *ex vivo* expansion of cells increases the risk of genetic instability and the occurrence of potential chromosomal abnormalities, since there exists a close relation between cell senescence and risk of transformation (Baxter et al., 2004; Rubio et al., 2005; Campisi, 2007). To limit this risk, the number of population doublings should be kept to a minimum. In addition, conventional karyotyping must be combined with fluorescence *in situ* hybridization (FISH) or comparative genomic hybridization (CGH array) to assess the genomic stability of scaled-up cell populations (Barkholt et al., 2013).

The control of the population identity into expanding cell cultures is generally realized by flow cytometry analysis of surface antigens. During the past decade, most of these controls have been realized in compliance with the recommendations of the International Society of Cellular Therapy (ISCT) (Dominici et al., 2006). However, it is today acknowledged that several markers initially proposed by ISCT for the positive characterization of MSC (for instance CD73, CD90, and CD105) are shared by several populations of cells including progenitor cells, mature fibroblasts or perivascular cells (Russell et al., 2010; Alt et al., 2011; Halfon et al., 2011; Al-Nbaheen et al., 2013; Lv et al., 2014).

## DP-CBMP uses

Over recent years, DP-CBMP were clinically tested with the aim to regenerate human craniofacial bone. DP-CBMP were implanted, in association with a collagen I-based sponge scaffold, in mandibular bone sockets in a phase I clinical trial (d'Aquino et al., 2009). Three years after DP-CBMP grafting, the tissue regenerated in the graft site was compact bone (Giuliani et al., 2013). Case reports of osteoradionecrosis treatment using DP-CBMP were also reported (Manimaran et al., 2014). The angiogenic, neurogenic and odontogenic potential of DP-CBMP was also successfully tested in preclinical studies (Gandia et al., 2008; Iohara et al., 2009; Sakai et al., 2012; Ishizaka et al., 2013). In addition, a phase I clinical trial is currently under progress to evaluate the DP-CBMP potential to regenerate the human dental pulp (Nakashima and Iohara, 2014). Despite these successes, potential applicability of DP-CBMP will be closely dependent on their final production cost and their large-scale clinical outcomes. In particular, a high cost-efficacy ratio would constitute a serious impediment for their routine use. Hence, it is necessary to have a clear overview and understanding of the complete value chain to try to reduce costs (Abou-El-Enein et al., 2013, 2014; Leijten et al., 2015).

Storage of cryopreserved cell-based products (cryobanking) over long periods of time offers unique opportunities to increase DP-CBMP applicability. However, similar to cell culture and expansion, cryopreservation is associated with infective, prion, toxicological and immunological risks owing to the presence of human or animal components and additives such as DMSO in the storage medium (Papaccio et al., 2006; Perry et al., 2008; Woods et al., 2009; Lee et al., 2012). Accordingly, xeno-free, defined cryopreservation media must be privileged.

DP-CBMP could also be used in biomedical research as components of bioassay kits to investigate the effects of drugs on dental pulp cells in a reproducible “humanized” system (Jurga et al., 2010; Leeb et al., 2011; Forraz et al., 2013). Such kits are reliable preclinical alternatives to animal models in the actual regulatory context. Assessment of the risks related to chemical products' use and screening or testing new therapeutic molecules are indeed extremely complicated and costly. The average costs to take a blockbuster drug to clinical trials are estimated to be around 1 billion euros. Furthermore, the accuracy of toxicological and preclinical studies greatly depends on the experimental animal models used for such evaluations. In particular, rodent species, widely use, are known to only partially mimic the human biological system. Development of DP-CBMP bioassay kits would offer a prime platform to successfully induce dentinogenesis, osteogenesis or neurogenesis *in vitro* (Zhang et al., 2006; Woloszyk et al., 2014; Jensen et al., 2015; Leijten et al., 2015).

## Proposals for a protocol with a more GMP compliant approach (Figure 2)

We recently proposed the use of impacted third molars between Nolla's developmental stages 5 (crown almost completed) and 7 (one third root completed). The presence of large, open apices in teeth without roots or with roots partially developed allows for an easy access to the pulp tissue and its gentle, atraumatic extirpation from the enamel/dentin shell with fine tweezers. It avoids the cell stress resulting from the crown-root mechanical separation with a drill or a clamp that is necessary for recovering pulps from teeth with more developed or complete roots (Perry et al., 2008; Takeda et al., 2008; Ducret et al., in press). Additionally, human dental pulp cells (HDPC) isolated at around the crown-completed stage displayed short cell doubling times and high growth rate (Takeda et al., 2008). We found similar results in our study. We also selected impacted teeth to minimize the risk of pulp tissue contamination and disease transmission by oral microorganisms (Nolla, 1960; Ducret et al., in press). This choice may enable to skip the step of sample disinfection performed with chemicals such as chlorhexidine or povidone-iodine/sodium thiosulfate (Perry et al., 2008; d'Aquino et al., 2009). When using PBS as a transport medium, we failed to detect any contamination in cultures of HDPC (*n* > 50 patients) during the isolation and expansion steps, contrary to others (Perry et al., 2008; Ducret et al., in press). This might be related to our selection of impacted teeth from young patients (13–17 year-old) that have never been in contact with the septic oral cavity, versus the selection of erupted ones from older patients (18–30 year-old) by those authors.

**Figure 2:**
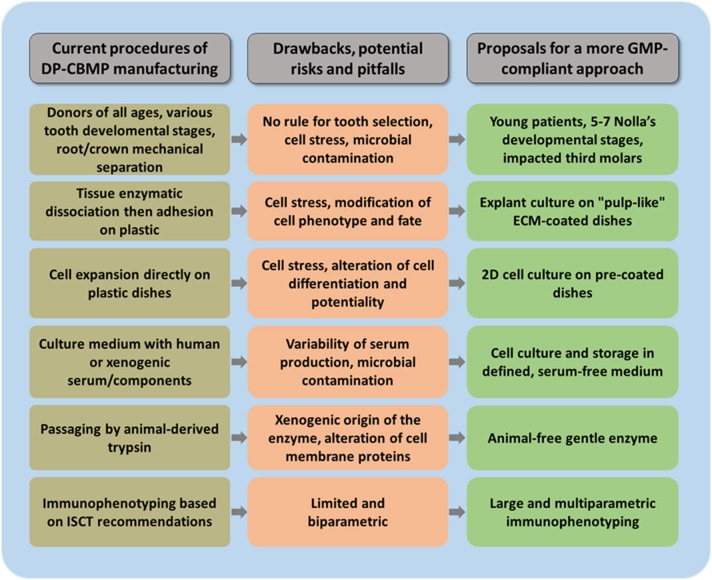
**Important steps during DP-CBMP manufacturing that require modification**. Major drawbacks, risks and pitfalls are highlighted for each one, and proposals are made for the development of more GMP-compliant procedures.

Regarding cell isolation, we used explant culture for recovering human dental pulp cells for clinical application. Each pulp sample was cut into about 20 explants that allowed for the harvest of a total of one million dental pulp cells after 14 days of culture (Ducret et al., in press). This result is in agreement with other studies reporting that, whatever the technique used (tissue dissociation or explant culture), 2 weeks of culture allow for the recovery of about 10^6^ cells from one third molar pulp (Eubanks et al., 2014).

We pre-coated the culture dish surface for cell isolation and culture with an equal mixture of human placental collagens I and III. This composition was chosen because they are the two most abundant collagens in the dental pulp extracellular matrix. Xeno-free dissociating reagents (such as TrypLe® or Accutase®) and xeno-free defined culture medium (such as SPE-IV® [ABCell-Bio, France], containing clinical grade human albumin, α-MEM, rhIGF-1 and rhFGF-2) are recommended for cell culture and passaging instead of the products commonly used (Carvalho et al., 2013; Ducret et al., in press). Moreover, cryopreservation of dental pulp cells in serum-free medium had no negative impact on cell doubling times and cumulative cell numbers (Ducret et al., in press). Although the viability of cells cryopreserved in serum-free medium was decreased compared to fresh cells, it is similar to that previously reported (Lee et al., 2012).

Future investigations are required for identifying more specific membrane markers for these cells. In our study, immunophenotypic analysis of 17 surface markers revealed that our dental pulp cell expanding population was made of mesenchymal cells, a percentage of whom expressed the mesenchymal stem cell/progenitor markers CD146 and MSCA-1. The number of cells expressing these markers remained similar from P1 to P4, suggesting that the cell fate was not significantly affected by our culture conditions. In addition, cell karyotyping by G-band analysis showed that this rapid expansion did not lead to genomic instability that would be potentially harmful for the recipient patient (Ducret et al., in press).

## Conclusion and perspectives

Recent successes in bone and dental pulp regeneration therapies carry the promise to use dental pulp-cell-based medicinal products in the near future. However, current strategies to manufacture DP-CBMP are not totally satisfactory since they do not comply with current international guidelines. New manufacturing standardized protocols, intended to increase efficiency, reproducibility and safety of these strategies, are urgently needed. Further investigations are also warranted to estimate the real benefit of DP-CBMP use compared to current therapeutic options and precisely determine the cost-efficacy ratio that risks being a major block for the large-scale clinical use of these cell-based products.

### Conflict of interest statement

The authors declare that the research was conducted in the absence of any commercial or financial relationships that could be construed as a potential conflict of interest.
